# A quiet harvest: linkage between ritual, seed selection and the historical use of the finger-bladed knife as a traditional plant breeding tool in Ifugao, Philippines

**DOI:** 10.1186/s13002-016-0124-9

**Published:** 2017-01-13

**Authors:** Kevin M. Murphy

**Affiliations:** Department of Crop and Soil Sciences, Washington State University, 113 Johnson Hall, Pullman, WA 99164-6420 USA

**Keywords:** Seed selectors, Rice, Ifugao, Finger-bladed knife, Transverse-harvest knife, Plant breeding

## Abstract

The transverse harvest knife, also commonly called the finger or finger-bladed knife, has been utilized by rice farmers in southeast Asia for many centuries. The finger knife persisted in many traditional cultures long after the introduction of the sickle, a tool which provided farmers with the means to execute a much faster harvest. Several theories in interpretative archaeology have attempted to account for this rejection of more modern technological innovations. These theories, which include community-based social organization ideas and practical reasons for the continued use of the finger knife, are presented in this paper. Here I suggest an alternate theory based on a re-interpretation of existing research and fusion of existing theories: the primary reason for the historical and continued use of the finger knife is for seed selection through a centuries old tradition of plant breeding. Though I accept the accuracy of the practical and community-based, socio-cultural reasons for the use of the finger knife put forth by other authors, I suggest that seed selection and genetic improvement was the driving factor in the use of the finger knife. Indeed, intricate planting and harvesting rituals, which both ensured and encouraged varietal conservation and improvement co-evolved with the use of the finger knife as the primary harvest tool due to its unique ability to aid the farmer in the art and science of seed selection. When combined with previous ideas, this interpretative theory, based on the connection between ethnoagronomy and material culture, may provide a more complete picture of the story around the persistence of the finger knife in traditional rice-growing cultures in southeast Asia. I focus my theory on the terrace-building Ifugao people in the mountainous Cordillera region of northcentral Philippines; however, to put the use of the finger into a wider regional context, I draw from examples of the use of the finger knife in other traditional cultures throughout the region of southeast Asia.

## Background

In this paper I utilize interpretive archaeology [1, 2] to present novel aspects to a theory which bridges the anthropological sub-fields of material culture and ethnoagronomy [3, 4]. I do not use original research in this manuscript, but rather use a discursive approach to focus on the intersection between the rice-based ethnoagronomy of the Ifugao people of the Cordillera Region of the Philippines and their material culture; specifically, the traditional use of the finger-bladed knife. Interpretation is a multivocal, ongoing process; there is no final and definitive account of the past and different interpretations of the same field are quite possible [2]. Indeed, archaeological interpretations are creative, likely to be suited to different purposes, and require the interpreter to take responsibility for his or her actions and interpretations [2]. Here, I argue that the finger-bladed knife was used primarily as a seed selection tool by the Ifugao to improve and diversify locally adapted highland rice varieties.

## Rice and culture of the Ifugao

The Ifugao people of Cordillera Region in north-central Luzon Province in the Philippines inhabit a steep, mountainous landscape approximately 17° north of the equator. Rainfall is abundant in this region, with 2000 to 3000 mm of rain per year falling on the mixed tropical montane forest and rice terraces ([5]: 1–4). Activities of the Ifuago are traditionally tied to agricultural management of ponded terraces (permanent cultivation) and swiddens (shifting cultivation), and ecological management of private forests (*muyong*) typically located above the primary farming locations. Food obtained through the farming of swiddens, primarily sweet potatoes, is used to supplement Ifugao diet, as a form of crop security if rice harvests are low or ponded terraces damaged ([5]: 1).

Monogamy is the rule of the Ifugao, who practice bilateral, consanguineal kinship, with secondary bonds of “neighborhood and propinquity” ([5]: 5). Inheritance of property, conflict resolution and decisions regarding agriculture, follows a primogeniture birth order ([5, 6]: 5). This inheritance rule allows both for the terraces and other agricultural land and private forests to remain undivided, and for rituals which emphasize Ifugao ancestor veneration to establish a clear connection between the living and the dead [6]. Ifugao live primarily in “hamlets” composed of families with terraces in the same vicinity, bound together by either kinship or common ecological concerns. Several dozen hamlets comprise an average “district”, each with or led by a *tomona*, the ritual leader who makes all district-wide agricultural decisions ([5, 6]: 6). The *tomona* owns a centrally located rice field, which is traditionally the first to be planted and harvested, and manages the property of this ritual field; in particular, the rice gods (*būl-uls*) and basket reliquary (*panu’būngan*) ([5]: 6).

Borrowing from the “house” concept of social organization [6–8] argues that the ritual agricultural field (*puntonaan*) acts as the central, connecting point of Ifugao social relationships and indeed becomes an emergent property that defines Ifugao social organization. The Ifugao have continuously grown rice in an intricate series of terraces for hundreds of years. The increased expansion of terraces throughout the Cordillera Region of the Philippines resulted in ever-greater demands on soil, land, and water resources, leading to a “self-organization” model of social organization, where increased resource pressures led villagers within adjoining settlements to share labor and limited natural resources such as ponded terraces, land for shifting cultivation and forest resources, and water [6]. This cooperative, self-organization is evident in the synchronization of various labor-intensive agricultural activities of communities within a watershed for activities including planting, weeding, pest control, irrigation, and harvest ([5, 6]: 1–39).

Movements and migrations of Austronesian speakers brought cultivars of rice and taro to the Philippines, and these two crops formed the basis of pre-historic food production in Luzon [9, 10]. The exact age of the rice terraces of the Ifugao region has been a matter of debate over the past 100 years. The pre-contact model put forth by Barton [11] and reinforced by Beyer [12] suggests that the terraces were 2000 to 3000 years old. This timeframe was based on calculations on the minimum amount of time it was projected to take to build terraces of this magnitude, and the pre-contact model is still the one most Filipinos adhere to today. The post-contact model suggests that the Spanish arrival to, and colonization of, Luzon in the 1600s expedited the movement of indigenous lowland groups to the Cordillera highlands, resulting in the construction of the rice terraces between 300 and 400 years ago [13–18]. Recent archaeological evidence suggests that the Little Ice Age, which increased aridity in the Cagayan lowlands while simultaneously increasing rainfall in the highlands, encouraged an earlier group of people who may have moved into the Cordillera highlands in the 13^th^ century [19].

The Ifugao people, due to their widespread construction and continued cultivation of their extensive system of rice terraces, are the most well-known of several minority ethnolinguistic groups in the Cordillera Region. Four clusters of terraces in the Ifugao region of the Philippine Cordillera were recognized by UNESCO as a World Heritage Cultural Landscape in 1995, and reclassified on the List of World Heritage in Danger in 2001. The terraces dapple the rugged landscape primarily across Ifugao and Mountain (formerly Bontoc) Provinces, but can also be found in the provinces of Apayao, Benguet, and Kalinga. In Ifugao Province alone there are an estimated 20,000 km of terrace walls, 7000 of which are composed of rock quarried from the mountainsides or alternatively carried up many hundreds of meters from the river bed below [5, 20].

Rice production holds a place at the center of the Ifugao worldview [5, 11]. Asian rice, *Oryza sativa*, is the staple crop for more than 50 % of the global population, and is the most widely grown crop species worldwide ([21]; World Rice Statistics, http://www.irri.org; FAOSTAT, http://fao.org). *O. sativa* was domesticated from *O. rufipogon* during the Neolithic era approximately 10,000 years BP, which gave rise to both the *japonica* and *indica* major variety groups [22, 23]. It is the tropical japonica subpopulation that is traditionally grown on hillsides in Southeast Asia [23, 24].

According to Conklin ([5]: 13–35), the rice growing cycle begins in much of the Cordillera (with notable exceptions) in the rainy season with terrace repair and formation and field preparation, typically performed by men, followed by rice planting by women (Phase I). As the dry season arrives, rice cultivation and weeding occurs, followed by harvest (Phase II). However, this farming cycle was based on the use of traditional rice varieties (in Banaue, collectively called *tinawon*) which were adapted to centuries old cultivation patterns. The traditional cycles were somewhat disrupted by the introduction of new varieties, which varied significantly in the number of days to reach harvest maturity, to the region ([25]: 42). However, adoption of new varieties from formal breeding programs outside of the Cordillera region was often resisted or very slow to take hold, due partially to their inability to fit within the Ifuago agricultural cycle. In addition to their importance in rice production in Ifugao, the terraces also serve as the primary location for cultural rituals.

Current challenges to the traditional Ifugao lifestyle includes the rapid influx of tourism, outmigration for lowland city and overseas employment, and the related decline of traditional farming practices and spiritual rituals, the latter often performed by the *mumbaki*, or local priest ([26]: 71). Efforts focused on the conservation of traditional rice varieties and historically sustainable farming practices are methods currently employed to help revitalize traditional rice production practices in Ifugao.

## Main text

### Traditional use of the finger-bladed knife

**Fig. 1:**
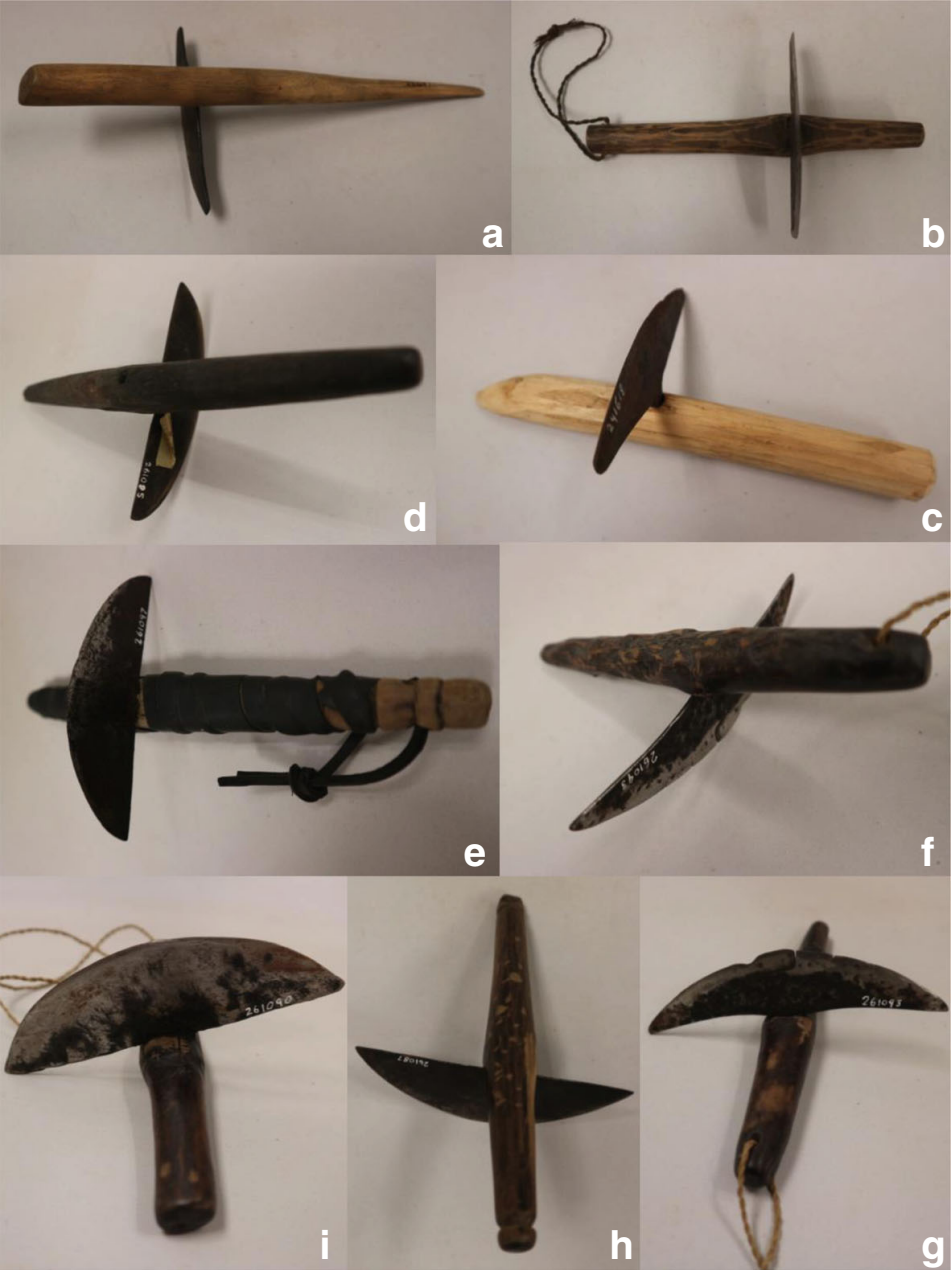
Photos illustrating variation in blade and handle shape of finger knives collected by Dr. Harold Conklin in the Philippines. Accession #’s and general information are as follows: **a** 241693; handle 25.5 cm, collected from Uma, Lubuagan kalinga-Apayao Province; **b**, **f**, **g** 261091; handle 19 cm, blade 11 cm, collected from Bayninan, Ifugao; **c** 241618; handle 19 cm, collected from Butbut, Kalinga Subprovince, Kalinga-Apayao Province; **d** 261085; handle 19.5 cm, blade 10.5 cm, collected from Bayninan, Ifugao; **e** 261097; handle 20 cm, blade 10.5 cm, collected from Bayninan, Ifugao; **h** 261087; handle 19.5 cm, blade 13 cm, collected from Bayninan, Ifugao; **i** 261090; handle 17 cm, blade 12 cm, collected from Bayninan, Ifugao. Photos: Kevin Murphy. The photos were taken with permission from the MET university

**Fig. 2:**
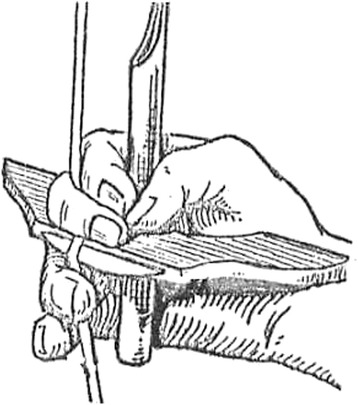
Illustration of the proper grip and use of finger knife. Credit: North Illinois University

Harvesting of rice is still accomplished in small, isolated areas of the Philippines with small handheld finger-bladed knives that likely resemble the very first harvest knives created [27] (Fig. 1). A small metal blade is fitted crosswise into a short piece of wood and the harvester holds the tool with the blade running transverse across the palm, fingers bent around the rice stalk beneath the panicle, and draws the stalk in toward the blade, severing the panicle from the rest of the rice plant (Fig. 2). Types of plants used for the handle range widely, from bamboo to hard woods such as mahogany. If metal for a blade was unavailable, farmers in the Philippines have been reported to use the sharp edge from the shell of a bivalve mussel, which could often be found in the irrigated rice fields [28]. The knives are called by various names throughout the Philippines due to the different languages and dialects spoken. For example, the finger knife is called ‘*rakem’* in Ilokano, ‘*rakam*’ in Isneg, ‘*lakom*’ in Kalinga’, and ‘*lakem*’ in Bontoc and Lepanto Kankanay [20].

Movillon and Schlosser [27] describe difference among traditional rice harvest knives most commonly used in the Philippines: the finger or transverse-bladed knife and the sickle. The sickle is a well-balanced tool with either a smooth or serrated blade shaped like a hook, which fits into a handle. Typically, the harvester will gather the rice stalks in a bundle in one hand and uses the other hand to cut the stalks close to the ground [27]. The time-saving benefits during rice harvest of a sickle over a finger-bladed knife is readily apparent. In the province of Nueva Ecija, Ilocano farmers continued for a time to harvest using the finger knife (*rakem* or *yatab*), while Tagalog farmers had long before become acculturated to harvesting rice using a sickle (*lincao or palot*). Here, the *rakem* was shown to require almost five times the number of hours per plot to harvest rice as the *lincao* ([29, 30]: p. 256)*.* In another report, the finger bladed knife took approximately 240–250 h to harvest a hectare of rice, compared to only 80–160 h per hectare when a sickle was used [27]. Sickles have the capacity to cut multiple stalks of rice at one time, whereas a finger knife is typically used on only one to two stalks at a time. This greatly increases the speed of harvesting when using the sickle.

Why would people choose to harvest with the finger knife if the sickle were available and a much faster tool for harvest? Why has the finger knife survived despite superior technology? Two interconnected theories, based on situational practicality and social organization and established cultural roles, provide compelling reasons for the continued use of the finger knife. The first theory suggests that while practically speaking, small knives are very labor-intensive, they are useful, and optimally suited for, certain situations (e.g. the harvest of one panicle at a time). The finger knife is superior to the sickle in harvesting individual panicles in an area where the rice has ripened unevenly. Taller, traditional varieties frequently found in the upland regions of Ifugao are better suited to harvesting with small hand-held knives [27]. In Nueva Ecija, Philippines, the use of the finger knife reduced shattering during harvest, thus conserving the greatest number of grains from the panicle, while almost eliminating the collection of extraneous weeds ([29, 30]: p. 256). The sickle is not as nimble, and weeds are often gathered up in the rice sheaves. Another practical benefit of the finger knife is its potential to salvage a rice crop that has lodged due to strong winds or heavy rains. The smaller sized bundles resulting from finger knife harvest allows for easier transport from field to storage facility, needs simply to be hung or flipped over to dry, and often does not require threshing until the rice is ready to be consumed due to the space saving size of the bundles. One bundle is often sized to meet the need for one family meal. While in storage, the bundles provide additional aeration to keep the rice seed from mold, sprouting, or spoilage [27].

The second common theory, often intertwined with practical issues detailed in the first theory, regarding the use of finger knives rather than sickles is due to the connection between traditional harvesting systems and moral principles associated with community employment and income sharing. Miles [31] argues that though the finger knife has no sacred significance among the Yao people of Thailand, it is critical because it promotes employment opportunities. Similarly, in Indonesia, the *bawan* harvesting system encourages farmers to open up their fields and invite villagers willing to participate in the harvest using traditional transverse-bladed knives (*ani-ani*). At the end of each day, payment is given on a percentage basis to each of the harvesters, resulting in a significant source of income and food to the rural and landless poor [27]. The Balinese painter Nyoman Meja depicts the use of the *ani-ani* in a social setting (Fig. 5).

When sickles replaced the centuries-old, traditional *ani-ani* knives on the Indonesian island of Java, rice production increased; however, so did poverty and malnutrition, primarily among women and children [32, 33]. It was suggested that the *ani-ani* was more than simply a tool for harvesting rice; because it was time-consuming and labor intensive compared to the sickle, it also served as a tool for a more village-wide, equitable distribution of rice [32, 33]. Larger farmers relied on landless villagers for harvest, thus providing them with a seasonal income, a share of the harvest, and a means of livelihood. With the introduction of the sickle, entrepreneurial harvest teams went from village to village to quickly perform the work that previously had been the responsibility of the landless poor. The rearrangement of social interactions that accompanied the change in harvesting technology from the *ani-ani* to the sickle strained formerly cordial social interactions and encouraged political unrest and the widening of the gap between the wealthy and the poor [32, 33].

Farmers in the Yao village of Pulangka in the mountains of north Thailand use the finger knife rather than the sickle to cut rice because it allows them to harvest during the wet weather of months that coincide with two of the less labor-intensive phases of opium production: seed broadcasting and primary weeding ([31]: 231). Rice panicles on plants of traditional landraces varieties often mature at different rates allowing for successive harvests on the same plant. The finger knife is ideal for cutting individual ripe panicles, allowing for careful and multiple harvests beginning at an earlier date than the rice could otherwise be cut with a sickle. The use of the finger knife enables the harvesters to reap the mature rice panicles from any given stand, while leaving the immature panicles behind. The Melaban Kantu′ in West Kalimantan, Indonesia, like other Ibanic groups, make a small early harvest (*nyuma*) of the earliest maturing panicles, followed by a second and third harvest [34].

Finger knives may also be ideal for cutting rice panicles in fields overcome with weeds. Another reason the Pulangka use the finger knife is due to their farming system which places a considerably lower priority than weeding than neighboring groups like the Karen, who devote approximately 1000 person hours per hectare to weeding ([35]: 111, 168). The Karen are able cut their rice with sickles because they harvest in relatively weed-free conditions [35]. The Pulangka on the other hand utilize the finger knife to cut rice in weedy fields, selecting this approach over a thorough weeding during earlier months; Miles [31] states that this is not possible with the use of the sickle. Dove [34] disagrees, stating that in his research with the Iban, they often harvest rice stalks and weeds together with a sickle, and then remove the weeds along with the chaff in their standard threshing and winnowing operations.

### Ritual, rice varieties and the finger knife in the Ifugao cultural system

Cultivation of *tinawon* landrace varieties is central to Ifugao social life and ritual practice; they are optimally adapted to local, high-altitude Cordilleran conditions, wet-farming systems and annual farming cycles ([36]: 283–284). For the Ifugao, a woman of prestige in the village ritually sows the first seeds of the planting season in her seedbed, after which she will confine herself to her house to fast for a day to mark the beginning of the rice planting season [37, 38].

*Tinawon* varieties have co-evolved around the yearly farming cycles and are indelibly linked to the extensive rituals of the Ifugao, which revolve around terraced farming systems. Because introduced, modern high-yielding varieties (HYV) were selected in centralized breeding research centers in the lowlands of the Philippines, commercial rice does not follow the same cycle as the *tinawon* varieties. The increasing use has disrupted both the ritual and ecological facets of Ifugao society ([36]: 283–284). Because the HYV varieties have a markedly different growth cycle and growth habit, the importance of ritual has diminished, and belief systems that were based on the local, culturally selected *tinawon* varieties are increasingly disregarded. For example, the fallow period that comes after the harvest season and lasts for several months depending on local cultural norms, is no longer a common agronomic practice. This is due to the shorter growing season of the HYVs, which many farmers utilize to plant a second crop. Without time for the soil to replenish itself, the consecutive and rapid cycling of rice has depleted soil nutrients after several years resulting low harvest yields ([36], 283–284).

The influx of higher yielding rice varieties negatively impacted Ifugao terrace ecology due to their reliance on synthetic fertilizers and pesticides. Mollusks, shellfish and fish that traditionally enhanced the Ifugao diet have largely been wiped out in the terraces due to toxicity caused by industrial chemicals ([36]: 284). The new rice varieties do not require year round inundation, the absence of which has led to an increase in abundance of *Polypheretima elongata*, a large earthworm whose tunnels weaken the terrace walls [39].

Harvesting comes at the end of the dry season and as the time for harvest time approaches, the elders place a taboo sign in the middle of the village and announce a period of rest to demonstrate respect for the soil and the rice plants. Seed selection is often the first harvest performed, typically by women. Once harvest begins, both men and women use the finger knife, “the indigenous harvesting knife made of steel mounted perpendicular to a wooden frame” [37]. The role of women as ‘seed selectors’ reflects on the high status of women in the society [37, 38]. The vital role of the elder female famers has lessened considerably with the introduction of commercial rice varieties, typically harvested with a sickle, as their extensive knowledge of traditional Ifugao *tinawon* varieties is no longer valued by the community [36]. This has also negatively impacted the role of elder women in their traditional roles of seed selectors in the Ifugao.

### Role of ritual in the use of finger knives across Southeast Asia

One of the earliest written accounts of the finger knife ([40] 1:112) suggests that its use in Java as “a grateful acknowledgement for an abundant harvest” originated in ancient times and that farmers were reluctant to harvest rice with other tools. If this tribute ceased to occur, it was commonly believed that the particular rice field would no longer continue to yield the farmer an abundant harvest ([40] 1:112). The tribute in this case is the arduousness of severing “each separate ear along with a few inches of straw” using the finger knife, even though other Javanese knives and reaping hooks available and in use at the time would be faster and more efficient ([31]: 227; [40] 1:112).

According to Skeat [41]: 58, the Malays adhere to the practice of using the finger knife out of “piety,” so that the “soul of the rice not being disturbed thereby.” Wilkinson ([42] 1: 604) states that the “wooden framework is held in the hand so as to hide the blade…. The underlying idea is that the rice grains shall not see the knife and that their vitality (*semangat*) shall not suffer through fright.”

Woensdregt’s [43] references to the use of the instrument among the ToBada’ of the Celebes stress that the knife “must not be transferred from one hand to the other because the soul of the rice might then shift to someone else’s field.” By dropping the tool a harvester may cause “the soul of the rice to take fright and there will be a small harvest.” The same author’s statements about the decoration of the implement typify many observations concerning the supernatural significance of the device’s shape and ornamentation. Additionally, Freeman reports:

“reaping a farm is a slow and protracted operation, for each panicle is plucked separately by hand….There can be little doubt that the reaping rate would be accelerated if sickles were used…but such a method is ruled out because of the reverential attitude with the Iban adopt towards their *padi*. In reaping with the ketap, the padi is taken as it were, unawares and with a minimum of shock or disturbance, and it is believed that if more drastic and unceremonious methods were introduced, the padii spirit would be likely to flee to other farms, and that as a result, the crop would be a poor one ([44]: 206–208).

**Fig. 3:**
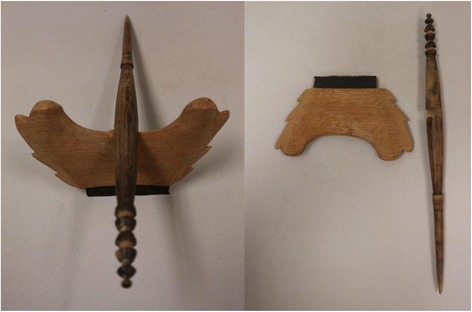
Finger knife with wooden handle carved in the shape of a bird. Collected from Tasek Bera, Pahang, Malasia. Accession #260102, Yale University. Photos: Kevin Murphy. The photos were taken with permission from the MET university

**Fig. 4:**
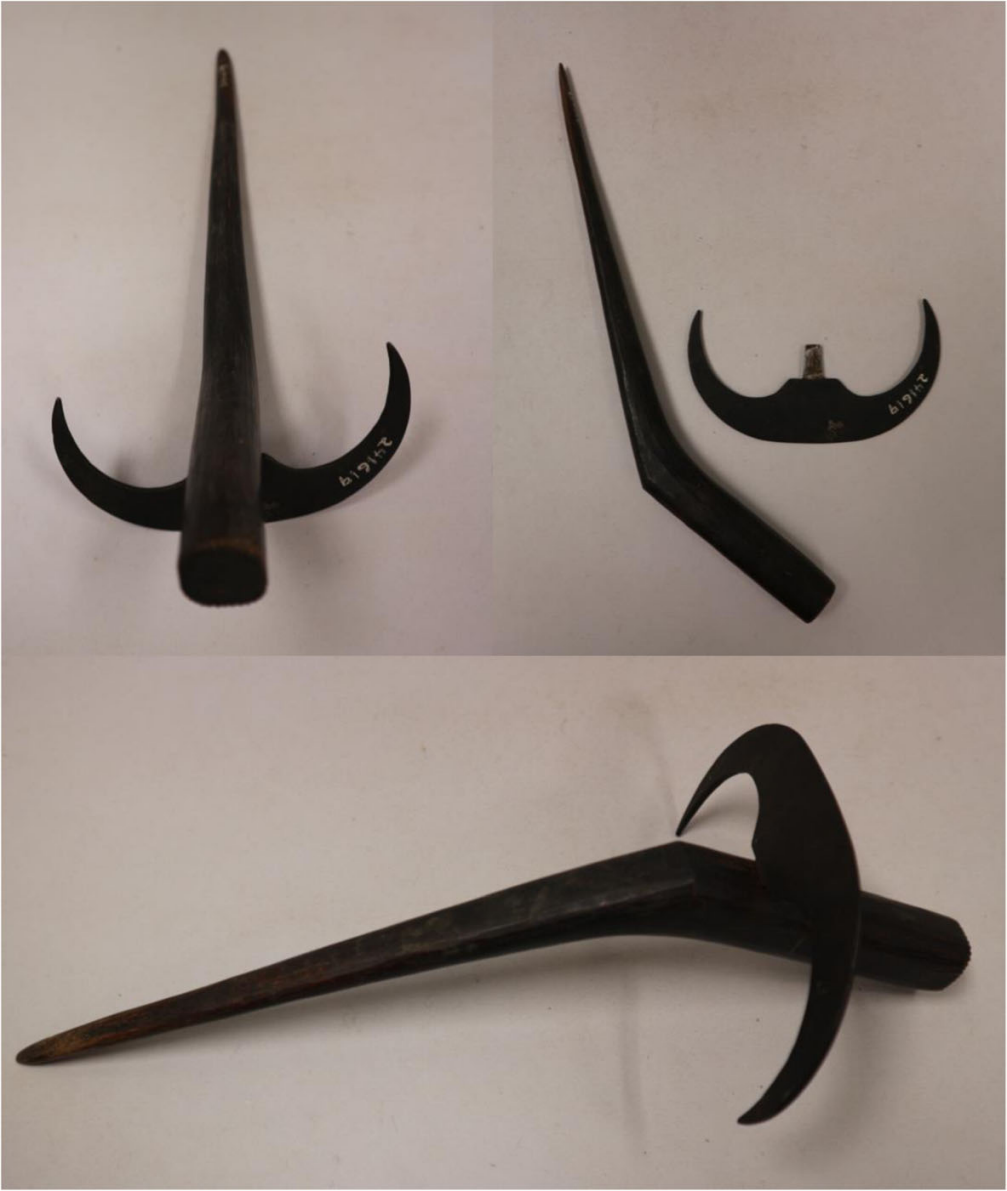
Finger knife with the metal blade cut in the shape of a bird. Collected by Dr. Harold Conklin in Butbut, Kalinga Subprovince, Kalinga-Apayao Province. Accession #241619, Yale University. Photos: Kevin Murphy. The photos were taken with permission from the MET university

Among the Ifugao, the tradition of using finger-bladed knives is deeply intertwined with the spiritual belief of a rice deity (Fig. 6). Rice gods (or *bul-ul*) are believed to be offended by harsh treatments of the rice plants, including through the use of a sickle, considered to be rough and alarming. If a sickle used, Ifugao tradition holds that the following season's crops will witness the displeasure of the *bul-ul* [27]. When the finger knife is quietly used, the rice plant does not become distressed by the approach of the harvesters, thus allowing for a painless and inconspicuous harvest before the rice plant know what is coming. As follows, farmers often would carve the knives to resemble birds, which the plants recognize and think that are simply coming to feed (Figs. 3 and 4). It is customary for harvesters to approach the rice plants quietly, whispering in tones and codes undecipherable by the rice spirits, and careful not to cast warning shadows as they harvest [27]. Woensdregt [43] suggests that the ToBada’ people of the Celebes carve the knife with horse and bird motifs “so that the harvest will proceed as swiftly as a horse may run and a bird may fly.” The shape and ornamentation of decorative finger-knife implements signifies the supernatural element of rice harvest culture and belief. Another slight variation along the same theme points to the idea that the setting of the small knife itself represents a bird ([45]: 94; [31]: 227), and that the rice plant spirits do not do not mind harvest from birds but do in fact “resent the brutal use of a large knife” [46].

In an instructive rice harvest-based Javanese myth, the gods Dewi Sri and Visnu incarnate themselves as birds to teach people that rice must be harvested in the same manner as birds peck at the crop ([47]: 273). Van Dapperen maintains that the finger knife has survived in Java because of this long-held respect for this rice harvest ritual since the first influence of Hinduism on the Javanese, approximately 2700 years B.P. Deviations from the bird motif have been reported among the Sarawak, whose finger knives include a brace that takes the form of a dragon (*naga*) ([48]: 409), a central symbol of Bornean theologies ([49]: 84; [31]: 227–8).

## Role of the finger knife in seed selection

Here I suggest an alternate theory: the *primary* reason for the historical and continued use of the finger knife is for seed selection through a centuries old tradition of plant breeding. Though I accept the accuracy of the practical and community-based, socio-cultural reasons for the use of the finger knife put forth by other authors, I suggest that seed selection and genetic improvement was *the driving factor* in the use of the finger knife. Indeed, intricate planting and harvesting rituals, which both ensured and encouraged varietal conservation and improvement co-evolved with the use of the finger knife as the primary harvest tool due to its unique ability to aid the farmer in the art and science of seed selection. Even in modern, highly technological plant breeding programs around the world today, the “art” of selection, based on the breeder’s intuition and experience, is considered vitally important to the release of new varieties; plant breeding is commonly defined as the art and science of improving traits and varieties of agricultural importance [50–52].

Utilizing relatively high levels of crop and varietal diversity have been shown that farmers logically and rationally exploit genetic diversity to allow crops to adapt to different environmental and cultural conditions, thereby decreasing risk, improving pest management, and providing for more stable yields and a varied diet [53–59]. Ethnoecological research also has shown that cultural values, memories and principles influence farmers’ decisions on what to grow ([60–62]; Rhoades and [60]). This extends to decision making regarding the selection, utilization and maintenance of traditional landraces over a long period of time, whereby farmers incorporate cultural traditions and practices that allow for the maintenance and continued improvement of food varieties ([55, 60, 63]). In addition, the development and use of site-specific tools and locally adapted agricultural systems have been used in traditional farming communities worldwide to repel pests, protect habitat, and conserve soil and water resources [64–66].

Skarbø [67] showed that in the highlands of Ecuador, the farmers who ate a higher proportion of traditional foods, spoke more Kichwa than Spanish in intra-family communication, and wore the traditional dress had higher levels of agro-biodiversity, including intraspecific diversity, on their farms. In particular, farmers who consumed more traditional foods were more likely to grow more total varieties and landraces of maize, tubers, fruit crops, beans, vegetables and herbs, indicating that the use of local food traditions plays an important role in the fate of the rich crop diversity of the region [67]. Furthermore, households in the study which preferred a diet with a high percentage of traditional foods also tend to grow the majority of these traditional foods rather than relying on the market. This suggests that maintenance of, and appreciation for, their cultural and agricultural heritage results in a stronger commitment to the cultivation and conservation of genetic and agro-biodiversity in their region [67].

Use of the finger knife is critical to the selection of each year’s seed-rice. The individual harvesting of rice panicles using traditional and/or indigenous cultural practices, have played a role in the development of the diversity of traditional rice varieties. The harvest of individual panicles allows the farmer/seed-selector to carefully select plants with desirable qualities, and use this seed as the seed for the following season [27]. Desirable characteristics will differ based on the regional microclimates, dominant diseases and pests, the most relevant agronomic, and seed quality properties. These could include such traits as resistance to disease or insect pests, plant height, panicle structure, degree of lodging number and number of fertile tillers, seed size and color, overall plant vigor and perceived grain yield. Ethnogastronomic seed quality traits such as taste, texture, cooking time, or stickiness of the rice [3] when cooked would be more difficult to differentiate at this time, but will be critical post-harvest selection criteria.

In the Philippines, the terms *penar* or *penal* from the Proto Nuclear Cordilleran dialect mean ‘rice grain used for seed.’ This term is used specifically for rice seed that is sown in a seed bed from which seedlings will be transplanted into a pondfield [20]. Estimates suggest that over 500 varieties of rice are adapted to the higher altitude (500 to 1600 m.a.s.l.), wet paddy, flooded farming system employed by the Ifugao ([26]: p. 71). The Kantu′ in West Kalimantan, Indonesia, do not randomly select any portion of the harvest for use as seed in the following year’s swiddens. Rather, they select their seed-rice during a special phase of the harvest (*ngami’benih*), each panicle being selected individually by the harvester for its visible, desirable characteristics [34].

Cooperation among Ifugao farmers is important because the organization of community labor and swidden field, rice terrace, and forest management is critical in order to minimize conflict from unequal access to natural resources like water. Ifugao cultural practices of inheritance rule designed to ensure the continuity of property ownership of the household; conflict resolutions that typically involve property claims, marriages and distribution of meat which illustrate that relationships are not bound to fixed territories all suggest that the Ifugao social organization is explained by the concept of the “house society” ([25]: 208). As such, the traditional agricultural practices in Ifugao have organizing principle. For example, the village ritual head (*tomona*) coordinates certain agricultural activities in ways to increase rice productivity, control water use, manage available labor which provide continuity to the village, or “house” ([25]: 210). The *tomana* owns a central plot (*puntunagan*) which is the traditionally the first to planted or harvested, and which serves as a signal to other villagers that they can begin planting or harvesting ([5]: 110). To put this practice into a more global context, at least within the region of Southeast Asia, we can look to the rituals and traditions associated with seed selection among the Baduy of West Java, Indonesia.

The Baduy people of the highlands of West Java still primarily grow traditional varieties of rice, despite an influx of high yielding varieties into the country. These rice varieties have been actively selected to match the varying local micro-environmental conditions, including a ecotypic diversity of soil type and fertility, exposure to sunlight, and water availability [68]. Interestingly, most rice consumed as food by the Baduy are high yielding varieties purchased in the lowland markets; the local landrace varieties are produced and maintained primarily for ritualist purposes involving their traditional swidden system [68]. The Baduy women, accompanied by their husband, carefully conduct selection for superior rice genotypes within each of the approximately 89 local landraces that are grown each year; this special process is called *dipasing*. After *dipasing* occurs, homogeneous bundles of panicles from each variety are selected, marked, and hung to dry on a bamboo pole [68].

A similar ritual found among the Baduy in West Java occurs just prior to planting, when the male head of a household prepares the *pungpuhunan* (the sacred place in the center of a swidden field). Two seeds of sacred rice, called the ‘rice mother’, are sown in the middle of the *pungpuhunan* [68], after which seven holes are planted with one landrace variety of sacred rice (*57-pare koneng*) inside the *pungpuhunan* and seven holes are planted with a different landrace variety of sacred rice (*53-pare ketan siang*) outside the *pungpuhunan*. A minimum of five sacred landraces are planted and kept separate by the planting of other non-sacred landraces (Fig. 7) [68]. This has the practical purpose of prevent cross-pollination or accidental mixing of the sacred landraces, thereby ensuring its purity. Outcrossing in rice ranges from 1 to 2 % in the domesticated, autogamous species *O. sativa* and from 7 to 56 % in *O. rufipogon*, the wild ancestor to *O. sativa* [69, 70], depending on floral characteristics, including stigma length, anther length and percent of exerted stigma [71].

## Conclusions

One of the primary reasons proposed for the extent of traditional rice diversity lies in the use of the finger knife. Grist ([46]: 59–60; c.f. [72] 167–8) and others suggest that an implement which cuts panicles individually allows the harvester and seed-selectors to notice and exploit variation. This heterogeneity in a rice field may include variation for traits such as plant height, panicle length, density or weight, straw strength, number and vigor of secondary fertile tillers, color in the leaves, straw and panicle, seed size and color, overall plant vigor and perceived grain yield, and resistance to disease or insect pests. In areas of wet-rice cultivation where sickles replaced finger knives, harvest proceeds more rapidly, thus diminishing the ability of the farmer to conduct selection, whether it be positive (taking seed from superior rice plants) or negative (removal of inferior plants from the harvested population). This can result in a loss of homogeneity and a slow decrease in the overall fitness of the rice population, often resulting in random and unselected mixed stands which produce lower yields [68].

Several theories in have been put forth to explain the seemingly unwarranted rejection of the superior technology found in the sickle compared to the finger knife. These theories, which include community-based social organization ideas and practical motives for the continued use of the finger knife, are completely valid and explain some of the reasons why finger knives were used for so long after the introduction of the sickle. However, I suggest that the most important reason that the finger knife remained in use for so long was due to the centuries-long co-evolution between the finger knife harvesting tool used by farmers and seed selectors and the planting and harvesting rituals which ensured both the conservation and the continual improvement of rice varieties.

## Abbreviations

HYV: High yielding varieties
UNESCO: United Nations Educational, Scientific, and Cultural Organization
